# Sharing Patient-Controlled Real-World Data Through the Application of the Theory of Commons: Action Research Case Study

**DOI:** 10.2196/16842

**Published:** 2021-01-19

**Authors:** Andreas Hager, Staffan Lindblad, Mats Brommels, Stina Salomonsson, Carolina Wannheden

**Affiliations:** 1 Upstream Dream AB Bromma Sweden; 2 Medical Management Centre Department of Learning, Informatics, Management and Ethics (LIME) Karolinska Institutet Stockholm Sweden; 3 Center for Observational and Real World Evidence Merck Sharp and Dohme Stockholm Sweden

**Keywords:** knowledge commons, learning networks, patient and family centered care, eHealth

## Abstract

**Background:**

Technological advances have radically changed the opportunities for individuals with chronic conditions to practice self-care and to coproduce health care and research. Digital technologies enable patients to perform tasks traditionally carried out by health care professionals in a more convenient way, at lower costs, and without compromising quality. Patients may also share real-world data with other stakeholders to promote individual and population health. However, there is a need for legal frameworks that enable patient privacy and control in such sharing of real-world data. We believe that this need could be met by the conceptualization of patient-controlled real-world data as knowledge commons, which is a resource shared by a group of people.

**Objective:**

This study aimed to propose a conceptual model that describes how patient-controlled real-world data can be shared effectively in chronic care management, in a way that supports individual and population health, while respecting personal data privacy and control.

**Methods:**

An action research approach was used to develop a solution to enable patients, in a self-determined way, to share patient-controlled data to other settings. We chose the context of cystic fibrosis (CF) care in Sweden, where coproduction between patients, their families, and health care professionals is critical in the introduction of new drugs. The first author, who is a lawyer and parent of children with CF, was a driver in the change process. All coauthors collaborated in the analysis. We collected primary and secondary data reflecting changes during the time period from 2012 to 2020, and performed a qualitative content analysis guided by the knowledge commons framework.

**Results:**

Through a series of changes, a national system for enabling patients to share patient-controlled real-world data to different stakeholders in CF care was implemented. The case analysis resulted in a conceptual model consisting of the following three knowledge commons arenas that contributed to patient-controlled real-world data collection, use, and sharing: (1) patient world arena involving the private sphere of patients and families; (2) clinical microsystem arena involving the professional sphere at frontline health care clinics; and (3) round table arena involving multiple stakeholders from different settings. Based on the specification of property rights, as presented in our model, the patient can keep control over personal health information and may grant use rights to other stakeholders.

**Conclusions:**

Health information exchanges for sharing patient-controlled real-world data are pivotal to enable patients, health care professionals, health care funders, researchers, authorities, and the industry to coproduce high-quality care and to introduce and follow-up novel health technologies. Our model proposes how technical and legal structures that protect the integrity and self-determination of patients can be implemented, which may be applicable in other chronic care settings as well.

## Introduction

### Moving From Self-Care and Health Care to Co-Care

In high-income countries, the top 1% of high-cost patients, predominantly patients with complex chronic care needs, account for almost one-fourth (24%) of health care expenditure [1]. Over half of high-cost patients are under the age of 65 years [1]. As the population with chronic illness grows, health care is under increasing pressure to use available resources efficiently, without compromising quality of care. In recent years, the necessity of stronger citizen involvement in health care service delivery has been emphasized using several related concepts, such as patient and public involvement [2], shared decision making [3], patient- and person-centered care [4,5], and coproduction [6].

Ostrom defines coproduction as “the process through which inputs used to produce goods or services are contributed by individuals who are not in the same organization” [7]. Von Thiele Schwarz [8] uses the term “co-care” to describe coproduction between patients and other actors involved in their care, such as health care professionals, and emphasizes the usefulness of digital technologies in facilitating the exchange of knowledge and experience between different actors. Digital technologies enable patients to perform tasks traditionally carried out by health care staff in a more convenient way, at lower costs, and without compromising quality [9]. For example, people measure their own blood pressure, adjust their insulin dose based on their own glucose monitoring, and perform intravenous antibiotic treatments at home, without the support or direct monitoring of health care staff.

### Learning Health Care Systems as Knowledge Commons

With technology literally in their back pockets, individuals produce large personal health information repositories that can be shared electronically [10]. In the context of this study, we use the term *patient-controlled real-world data* to describe personal health-related data that are controlled by patients. Patient control implies that patients, in line with the General Data Protection Regulation (GDPR) [11], determine the purposes and means of the processing of their personal data, which does not prevent other parties from also being authorized as controllers, by the patient or by law. If adequately utilized, patient-controlled real-world data collected by patients and informal caregivers, in combination with health care collected data, can fuel the development of learning health care systems [12,13]. The information and knowledge that are shared in such collaborative systems can be conceptualized as *commons* [14]. The concept of commons refers to resources that are shared by a group of people and has its roots in the study of shared natural resources (eg, land and water) that are subject to social dilemmas such as competition, freeriding, and overharvesting [15]. In the 1990s, the concept gained application in the study of distributed digital information and knowledge, which Hess and Ostrom describe as knowledge commons [15].

In knowledge commons composed of personal health data, two social dilemmas in particular have been highlighted, namely disempowerment and enclosure [16]. Disempowerment of the individual in relation to the information industry has been described as a problem of unsustainable data practices that can also be labeled as a “privacy harm” resulting from uncontrolled data analytics [16]. As we “*bleed* data when moving a mouse cursor,” large industry actors like search engines gain power over us, which they can use to nudge us into certain behaviors (eg, by directed advertisements) [16]. The enclosure of data refers to the exclusive access to data by certain actors in the information industry, while not allowing others to profit from it, which is a type of appropriation problem [16]. The individual patient is the only source of real-time observations and experiences that span a lifetime of chronic illness. Several stakeholders have large resources and a mission to collect and act on patient-reported real-world data (eg, health care funders, national authorities, and the industry). Disempowerment and enclosure are prevalent social dilemmas in the current system of data flow between stakeholders, which hampers the effective development, introduction, and evaluation of new health technologies.

The knowledge commons is a useful framework to identify such problems based on how information is produced and used by different stakeholders, and to guide the development of suitable solutions to address these, for example, by the definition of social rules and legal mechanisms that enable individuals to effectively share ownership and control of resources [17]. To the best of our knowledge, strategies to explore and effectively manage such dilemmas in the health care and life science industries are limited. Therefore, in this study, we use the knowledge commons framework to explore how to develop adequate technical and legal structures for sharing patient-controlled real-world data.

### Aim

This study aimed to propose a conceptual model that describes how patient-controlled real-world data can be shared effectively in chronic care management, in a way that supports individual and population health, while respecting personal data privacy and control.

## Methods

### Study Design

We used an action research approach that is suitable when the purpose is to bring about change in real-world environments [18]. Action research is focused on solving a specific problem in a specific context. Contribution to both practice and research is achieved through the combined expertise of practitioners and researchers, which mandates a collaborative research approach [19]. While no specific method of data collection is specified, a variety of generally qualitative methods may be applied, including journal keeping, document collection, and case studies [18]. In the following subsections, we first describe the chronic care context that was chosen to address the aim of this study. Thereafter, we describe the action research process and the data collection and analysis methods that were used.

### Chronic Care Context

We selected the context of cystic fibrosis (CF) care in Sweden. This context was chosen because CF care and its improvement are strongly dependent on coproduction between patients, their families, and health care [20,21]. CF is a life-limiting recessive genetic disorder that is usually diagnosed within the first few years of life, affects several organs, and leads to chronic infection and inflammation in the airways [21]. Treatment involves drug therapy, daily self-management (eg, airway clearance and physical exercise), and support by multidisciplinary care teams [22]. The recent development of disease-modifying drug therapies has substantially improved the prospect of effective treatment for CF [23]. The availability and use of solutions that enable patients and their families to share patient-controlled real-world data are critical to support the introduction and follow-up of these new therapies. In Sweden, there are approximately 700 CF patients, and about 95% of them are included in the national CF quality registry where care process and outcome data are reported for benchmarking and quality improvement [24].

### Action Research Process

The main change agent in the action research process was the first author (AH) who is a lawyer and parent of children with CF, as well as a driver in the development of mobile patient support systems (PSSs) to support patients and their families in self-care and communication with health care. To adequately explore the development of a new infrastructure for sharing of patient-controlled real-world data in the context of CF care in Sweden, the researchers collaborated with AH, combining their contextual and theoretical knowledge to contribute to problem-solving and knowledge generation. Action research is cyclical by nature, which means that changes are continually evaluated, leading to incremental improvements. While the change process itself is not the focus of this study, we analyzed the resulting changes that enabled patients to self-determinedly share patient-controlled real-world data to different stakeholders in CF care. The action research process was guided by the knowledge commons framework.

### Knowledge Commons Framework

The knowledge commons framework defines concepts that are central to understanding knowledge as a shared resource. A central concept is the *action arena,* which consists of the individuals and organizations who make decisions that affect patterns of interactions and outcomes in a knowledge commons [25]*.* To understand the complex nature of information and knowledge sharing, the knowledge commons framework makes a distinction between *ideas*, *artifacts*, and *facilities* [26]. An *idea* is an intangible representation of information in an individual’s mind. For example, someone’s experiences or thoughts that have not yet been translated into natural language. An *artifact* is a discreet and nameable representation of an idea or a collection of ideas. For example, an electronic health record is a digital collection of ideas about an individual’s medical history. Digital artifacts can be used concurrently by multiple users and are stored in information facilities. A *facility* consists of the software and hardware that stores artifacts and makes these available to users. The distinction between ideas, artifacts, and facilities is important for the specification of legal structures in commons. To ensure sustainable use of a shared resource, different sets of rules need to be applied. One set of rules regulates access to the facilities of commons and another set of rules regulates the contribution and withdrawal of artifacts to and from the commons. Seven types of property rights have been identified as relevant in rule setting in digital information commons [25]. These concern the right to access a physical area (*access right*); the right to contribute to content (*contribution right*), extract information (*extraction right*), and remove information (*removal right*); the right to regulate use patterns or make changes to a facility, for example, by adding new functionalities to an application (*management/participation right*); the right to determine access, extraction, and removal rights (*exclusion right*); and the right to sell or lease extraction, management/participation, and exclusion rights (*alienation right*).

### Data Collection and Analysis

We collected primary data in the form of formal documents (project plans, meeting notes, technical specifications, contracts, and user manuals) and secondary data in the form of scientific publications [27,28], a master’s thesis [29,30], and a conference abstract [31] reflecting the design, development, implementation, and adaptation of changes during the time period from 2012 to 2020. These data were corroborated by the personal experience of AH who was a driver in the change process.

We performed a qualitative analysis resembling a directed content analysis [32], guided by the knowledge commons framework. Two authors (AH and SL) read through the collected documents and identified text that matched any of the knowledge commons concepts described above, that is, descriptions of action arenas, facilities, artifacts, and ideas, as well as descriptions of rules or property rights that were applied to protect personal privacy and control. Text that matched these concepts was extracted, labeled, and categorized. The relations between concepts were explored and summarized into models that visualize these relationships. The models were refined in discussions among all the coauthors, resulting in the conceptual model presented here.

## Results

### Overview

The first section briefly summarizes the main developments that resulted from the action research project and allowed patients and families to share patient-controlled real-world data with different stakeholders in CF care. Where applicable, references to relevant publications that report these changes are provided in parentheses. The second section presents our analysis of the implemented changes, which is presented as a conceptual model consisting of the different action arenas that we identified and the patterns of patient-controlled real-world data flow between them.

### Main Developments

In 2014, a mobile patient-facing application, Genia PSS, was introduced at one of the four national CF centers [27]. The Genia PSS was designed to help patients and their families keep track of patients’ health and self-care activities, and to facilitate communication, relationship building, and shared decision making together with their care team. Using the Genia PSS, patients could track and share their own health observations with their care teams. A health information exchange was developed for enabling import of shared data from the Genia PSS into the national CF quality registry.

In late 2015, a new and very costly disease-modifying CF combination therapy, lumacaftor/ivacaftor, was approved for market introduction in Sweden [33]. Not all patients eligible for therapy are responders, and it requires structured follow-up and evaluation of treatment effectiveness, following the national managed introduction process [34]. In 2016, the Sweden CF Coalition was formed as a collaborative learning network involving a variety of stakeholders, comprising representatives from all four national CF Centers, the National Cystic Fibrosis Association, the CF Working Group of the Swedish Society of Medicine, the National Quality Registry Development Group, and the Genia PSS Development Group. Author AH had a role as secretary, and SL served as an advisor. The CF Coalition defined their purpose as follows: “[to make] it easier for everyone – persons with CF, families, clinicians, researchers, and others – to work together to improve health, care, and cost of illness for people with CF” (Sweden Coalition Charter, May 21, 2016). In 2017, they formed a Coordination Group to take on the specific task to implement a national system for the orderly introduction and follow-up of lumacaftor/ivacaftor, using patient-collected and health care–collected real-world data [31]. Through the establishment of a multistakeholder learning network and implementation of new health information exchange services to the national quality registry, the Genia PSS enabled the use of patient-controlled real-world data to support quality improvement, research, and the orderly introduction and follow-up of new CF therapies. In 2018, when the treatment follow-up routines were in place, lumacaftor/ivacaftor was approved for subsidy in Sweden and introduced on the market. At the time of writing, each of the four national CF centers was connected to the Genia PSS, which, in October 2020, had about 240 active users with CF in Sweden.

### Three Arenas for Collaboration

**Figure 1:**
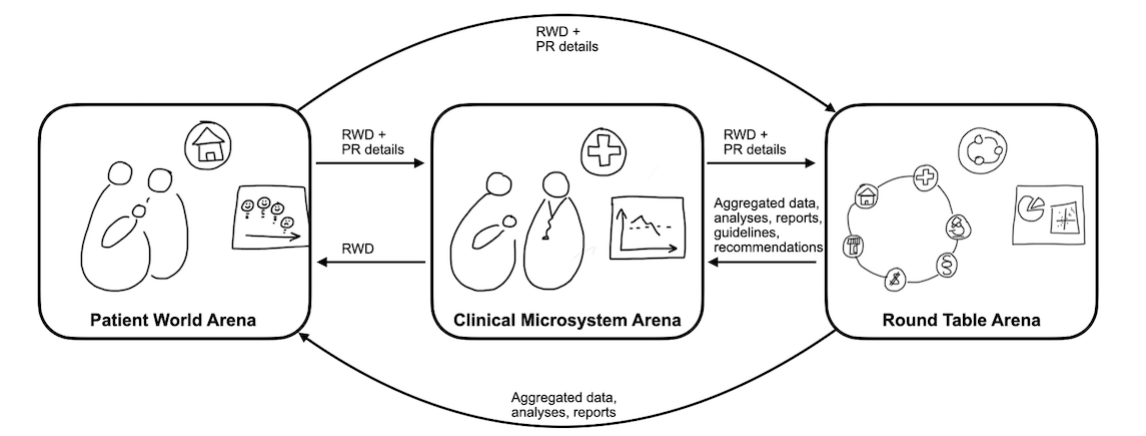
Three central arenas in the collection, use, and sharing of patient-controlled real-world data (RWD). PR details: specification of property rights that translate into use restrictions.

The analysis resulted in a conceptual model describing how patient-controlled real-world data can be used and shared across the following three action arenas in CF care: patient world arena, clinical microsystem arena, and round table arena (Figure 1). In the following sections, we describe the three arenas and the property rights that were specified to manage patient-controlled real-world data.

#### Patient World Arena

The *patient world arena* comprises the private sphere of patients and families living with CF. Using the terminology introduced by Kleinman, the actors in this arena cope with *illness*, *illness problems*, and *illness behavior* [35]. The patient and family together observe and experience health and well-being in the presence of *illness*, as well as the principal difficulties that symptoms and disabilities create for the patient (*illness problems*). They make judgements about how best to cope with the distress and with the practical problems in daily living, including initiating treatment, handling self-management, and deciding when to seek care from professionals and others (*illness behavior*).

##### Collection and Use of Patient World Artifacts

The Genia PSS enables actors in the patient world arena to capture nonphysical ideas of personal importance about illness, illness problems, and illness behavior, and generate physical artifacts that can be communicated and shared. Artifacts may represent assessments (eg, perceived health benefit from antibiotic treatment), memos (eg, a picture of a run in the forest), or narratives (eg, a treatment diary). The use of additional facilities, such as a blood glucose monitor, a pulse sensor, a scale, and a spirometer, may be necessary to capture health parameters of importance (eg, blood glucose levels, pulse, body weight, and lung function). Personal health data can also be imported for private use in the patient world arena from facilities used in other settings, such as electronic health record (EHR) and pharmacy systems. Collected and imported patient-controlled real-world data are stored in a personal database that can be made available via interfaces with patient-determined members of the patient world arena, such as family members and close friends.

##### Sharing of Patient World Artifacts With Other Arenas

Health information exchanges were implemented to support sharing of patient-controlled real-world data with other arenas, for example, with health care professionals prior to visits [28]. The sharing of artifacts with other facilities requires mutual agreement and adherence to information standards and terminologies (semantic interoperability), as well as contractual obligations and data protection legislation (legal interoperability). The Genia PSS has implemented a *previsit form* data structure to enable the sharing of patient-controlled real-world data with the clinics via EHR systems and the national quality registry.

##### Property Rights

The patient world arena represents a private domain within which patient-controlled real-world data are processed with no direct connection to professional or commercial activity. Property rights have been iteratively refined to safeguard personal data privacy and control (Textbox 1). The patient determines who has access to the Genia PSS as a member, the level of interaction with the system, and what patient-controlled real-world data to share. Especially for younger pediatric patients, parents serve as proxies by documenting daily observations about their child [28].

**Seven property rights for patient world arena users of the Genia patient support system.**

Access: Two defined user roles have access to the Genia patient support system (PSS) facility: *Patient* (individuals with cystic fibrosis) and *Member* (the innermost network of trusted persons as defined by the patient).

Contribution: Patient and Member users have the right to contribute with data to the patient account they are linked to.

Extraction: Patient and Member users have the right to extract data. Extraction is supported by functionalities in the Genia PSS. The user first selects the data items to extract and generates a report that can be shared. Extractions are logged.

Removal: Patient and Member users have the right to remove data from the patient account they are linked to.

Management/participation: The right to regulate general use patterns and make changes to the Genia PSS is retained by the service provider. Patient and Member users can personalize their individual patient account and activate available feature bundles.

Exclusion: Patient users determine who has the right to access, contribute, extract, and remove data, and how these rights may be transferred to Member users. Certain exclusion rights have been transferred to the service provider under the user agreement. For example, to maintain the integrity of the user’s private domain, the service provider has decided to limit the facility to private use, excluding professional or commercial use. This ensures the applicability of legislation that protects the freedom of the domestic domain, medical device regulations, and consumer protection regulations.

Alienation: Alienation rights to the Genia PSS facility (application and servers) have been retained by the service provider, that is, the right to sell or lease management/participation rights. The Patient user has alienation rights to the data, that is, to sell or lease extraction and exclusion rights and how those rights may be transferred.

#### Clinical Microsystem Arena

The *clinical microsystem arena* comprises the professional sphere at health care clinics. Clinical microsystems are the small, functional, front-line units that provide direct clinical services to patients, that is, the place where health care professionals and patients meet. Health care professionals manage patient care by following clinical practice guidelines that are agreed upon by the professions involved and accepted by their patients. In Sweden, patients meet with their care teams for quarterly check-ups at one of the country’s four CF centers, or, for patients who live outside commuting distance, at one of over 60 care centers that collaborate in “shared care” with a designated CF center. All annual check-ups are carried out at CF centers, which represent different clinical microsystem arenas. They are essential building blocks of the larger health system that supports CF patients and the university hospital organizations they are part of.

##### Collection and Use of Clinical Microsystem Artifacts

Actors of the clinical microsystem arena collect and store patient data in the various EHR systems. If granted access by patients, they may also import patient-controlled real-world data from the Genia PSS. The data are accessed through an export interface in the Genia PSS and imported into a specific module of the EHR, which will here be described as the Genia decision support system (Genia DSS). Once imported into the Genia DSS, the data can be processed according to the rules of the clinical microsystem. At each of the four national CF centers, functional features for processing patient-controlled real-world data that support daily clinical practice and care at home were developed and tested iteratively through small-scale design cycles with health care professionals and patient and family representatives.

##### Sharing of Clinical Microsystem Artifacts With Other Arenas

The Genia DSS is interoperable with the Genia PSS, as well as the national quality registry through a health information exchange service. To share patient data stored in the EHR system with the patient or the national quality registry, data export areas have been implemented in the Genia DSS, similar to those in the Genia PSS. The export area of the Genia DSS hosts data that have been approved and released for sharing and has an interface that can be accessed by the Genia PSS. For data that are shared with the national quality registry, deidentification of sensitive personal data (eg, through pseudonymization) may be required prior to making data accessible to quality registry users, so that individuals cannot be identified.

##### Property Rights

The clinical microsystem arena practices under a number of laws, regulations, and guidelines (eg, health information technology and patient safety legislation, and clinical practice guidelines). Therefore, the rules that govern health care professionals’ use of patient-controlled real-world data must be aligned with these. Property rights that have been applied in rule setting in the Genia DSS for protecting patients’ privacy and control are described in Textbox 2.

**Seven property rights for clinical microsystem arena users of the Genia decision support system.**

Access: The Genia decision support system (DSS) can primarily be accessed by health care professional (HCP) users (certified health care professionals). Patients have no user accounts in the Genia DSS, but they have access to designated computer terminals at the cystic fibrosis (CF) centers, with a data input and dashboard module. Also, HCPs can present patient facing dashboards to them during clinical visits.

Contribution: HCP users have the right to contribute data to their patients’ records in the Genia DSS. Patients can contribute data through patient-facing input modules at CF center computer terminals.

Extraction: HCP users have the right to extract data, which are constrained by rules in the clinical microsystem, patient consent, as well as guidelines regarding data privacy and patient safety. Extraction is done through standardized report formats. Extractions are logged for auditing purposes.

Removal: Removal rights are restricted in ways similar to accounting systems. Data can be modified and deleted from the user’s view in the Genia DSS. For data to be irreversibly deleted from the system, special legislation might apply, such as a Swedish regulation that requires a court decision initiated by the patient. Removal activities are logged for auditing purposes.

Management/participation: The right to regulate general use patterns and make changes to the Genia DSS is retained by the service providers. These rights are influenced by design recommendations and the given health care context, as well as potential constraints by other systems in use.

Exclusion: Generally, the head of the clinic has exclusion rights.

Alienation: Alienation rights to the Genia DSS facility (application and servers) have been retained by the service providers, that is, the right to sell or lease management/participation rights. The controller of data (in general, the clinical department) has alienation rights to patient data artifacts, that is, to sell or lease extraction and exclusion rights and how those rights may be transferred.

#### Round Table Arena

The *round table arena* can be defined as a multistakeholder collaborative learning network that works toward common goals. The members of the round table arena may represent individuals (eg, a patient) or institutions (eg, a hospital, authority, or industry actor). A round table arena that includes stakeholders with varying expertise and experience provides distinct advantages, not least of which is the ability to gather and share lessons in real time, contributing to the generation of intellectual and social capital. The Sweden CF Coalition and its Coordination Group are examples of round table arenas that require collective action, that is, the efforts of more than two individuals, to reach their purpose. To implement a system for supporting the orderly introduction and follow-up of lumacaftor/ivacaftor, good coordination is required with national health authorities and councils involved in decisions regarding the introduction and subsidy of the new therapy.

##### Collection and Use of Round Table Artifacts

**Figure 2:**
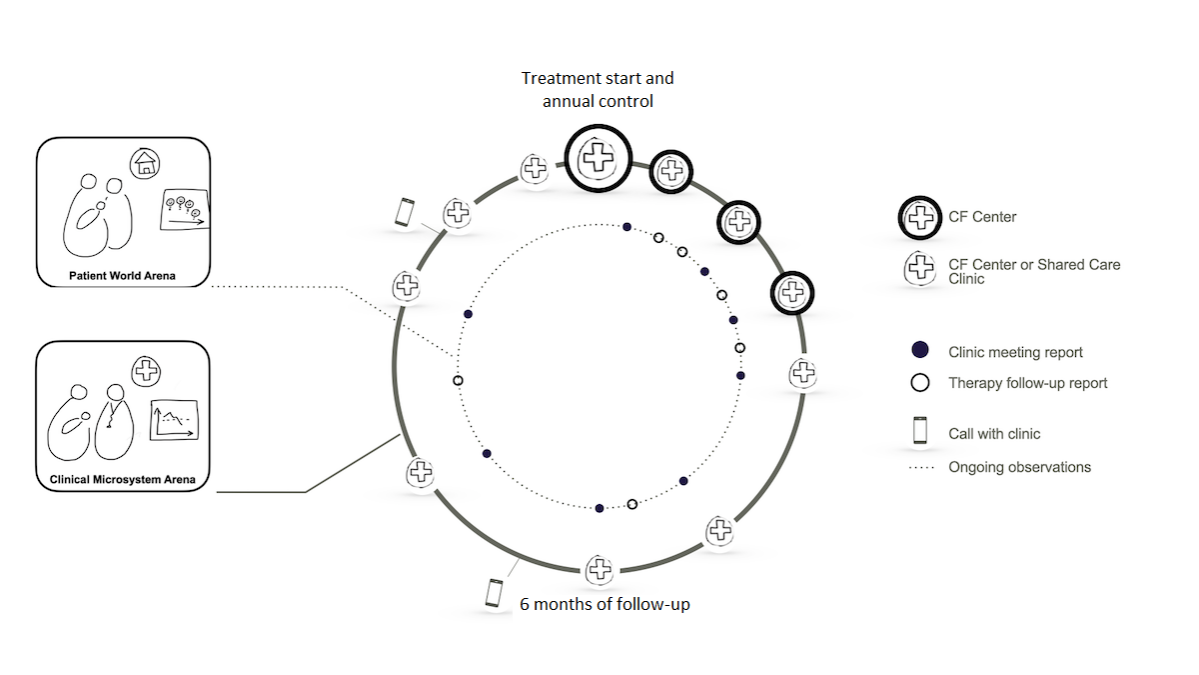
First-year customer journey with lumacaftor/ivacaftor therapy (an idealized flow with a new therapy linking the patient world and clinical microsystem arenas).

With data from the national quality registry as a shared resource, as well as the personal expertise and experiences shared by its members, the Sweden CF Coalition aims to set up crucial conversations among stakeholders, so they can learn about new ways to generate optimal health and high-value care. To implement a national system for the orderly introduction and follow-up of lumacaftor/ivacaftor, changes to existing facilities that hold patient-controlled real-world data were necessary. This involved negotiations on what core data sets could be extracted from the Patient World and Clinical Microsystem arenas. New modules for lumacaftor/ivacaftor follow-up visits with indicators for follow-up and evaluation of the therapy were implemented in the Genia PSS, Genia DSS, and national patient registry. The health information exchange service was modified to enable ethically and legally viable sharing of data across arenas. These modifications enabled the provision of a shared resource to support clinical decision making, quality improvement, administration, and research. The system was implemented in the summer of 2018 upon national approval of market access to lumacaftor/ivacaftor (Figure 2).

##### Sharing of Round Table Artifacts With Other Arenas

Actors in the Sweden CF Coalition share their collaboratively generated ideas through analyses, reports, and clinical guidelines. These artifacts are made available to selected stakeholders through creative commons or fee-for-service agreements in accordance with industry standards and national ethical platforms, or generally made available to the public. The information and knowledge that are produced by the round table arena can be described as *common-pool resources*, which means that they are controlled by stakeholders within the Sweden CF Coalition.

##### Property Rights

In contrast to the patient world arena and the clinical microsystem arena, where conversations about health concern identified individuals, actors in the round table arena have access to data representing deidentified individuals or a nonidentified collective of individuals [36]. Based on different round table arena actors’ roles and affiliations to other settings, the information they contribute to and extract from the facilities they have access to will be governed by different property rights. The data made publicly available by the Sweden CF Coalition are from the national patient registry for CF, which serves as an *open-access common-pool resource* where no one has the legal right to exclude anyone from using the resource, as long as it is in conformance with general legal requirements.

## Discussion

### Principal Findings

This study used an action research approach to propose a solution for enabling patients to effectively share patient-controlled real-world data in CF care. The case that was presented is an example of coproduction in and beyond health care services, where patients contribute to the orderly introduction and follow-up of new therapies, while retaining control of their data. We identified the following three types of action arenas where patient-controlled real-world data are collected, used, and shared: patient world arena, which comprises the private sphere of patients and their families; clinical microsystem arena, which comprises the professional sphere at health care clinics; and round table arena, which comprises stakeholders from multiple spheres with complementary expertise. The relationships and information exchange between these arenas were illustrated in a conceptual model, which we suggest can be used as a tool to analyze current chronic care systems and as a guide for the development of solutions for patient-controlled real-world data sharing.

### Comparison With Prior Work

In the following sections, we discuss our results in relation to prior work. We discuss the conceptualization of the three arenas in our model, the sharing of patient-controlled real-world data, and, finally, the applicability of the model in other chronic care settings.

#### The Three Arenas

The patient world arena is a central building block of the conceptual model presented in this study. In line with well-known concepts, such as *patient-centered care* [4], it emphasizes the patient’s experience and expertise on living with a chronic condition. Our model acknowledges that patients may involve actors outside the health care organization in their self-care. Thus, the patient world arena describes the patient’s domestic domain where laws and regulations of the health care setting and general privacy directives do not apply [37]. While each patient world arena has its own (likely implicit) social rules and norms for how data are captured, used, and shared, our model identifies some general property rights that may be applied.

The concept of the clinical microsystem, in contrast to the patient world, is well established. Nelson et al [38] described the clinical microsystem as “the local milieu in which patients, providers, support staff, information, and processes converge for the purpose of providing care to meet health needs” [38]. It can be seen as an arena for coproduction between the patient and his or her self-determined network and the formal care team, aimed at promoting, improving, or maintaining individual health. As Ostrom notes [7], “technologies in use must generate a complimentary production possibility frontier […] rather than merely a substitutive one. […] When coproductive inputs are legally owned by diverse entities and complements, synergy can occur.” Thus, while the clinical microsystem may be embedded in the health care organization, the patient represents an own entity when appearing as an actor and guest in the clinical microsystem arena. Here, the specification of property rights helps to clarify the ownership and control of the different inputs to the system, where patient-controlled real-world data that are produced in the health care setting or at home are controlled by the patient, but can be shared with the care team for use in the patient’s individual care.

The concept of the round table arena builds on the previous work by Lindblad (SL) et al [36,39] on registry-based learning networks, as well as previous research on collaborative chronic care networks [40-42]. It differs from the other arenas in a number of aspects. First, while the patient world and clinical microsystem arenas deal with coproducing strategies to maximize individual health, the round table arena deals with coproducing strategies to maximize population health. The Sweden CF Coalition that was analyzed in this study can be described as a double-loop learning system [43] that engages a multistakeholder group in following up and discussing the adequacy of new therapy practices and related guidelines, based on an aggregation of data from the patient world and clinical microsystem arenas. It deals with quality improvement on a national level. Second, while the patient world and clinical microsystem arenas are found within the boundaries of the domestic domain and health care organization, respectively, the round table arena crosses organizational boundaries. Third, while the purpose and stakeholders of the patient world and clinical microsystem arenas are well known to the patient, the round table arena may be formed for a variety of purposes and in a variety of settings. The patient who contributes with data may not know the stakeholders and may not gain any direct benefit that contributes to his or her individual care, at least not immediately. Thus, communication of purposes and agreements on the terms of patient-controlled real-world data management may be all the more important.

#### Sharing of Patient-Controlled Real-World Data Between Arenas

In this action research case study, we identified several different facilities that were used by stakeholders to share and access patient-controlled real-world data (the Genia PSS, Genia DSS, and quality registry). Sweden has long experience with clinical quality registries that allow patient-valued health outcomes and research to be coproduced between different stakeholders and provide open access to quality registry data for quality improvement and benchmarking purposes [36,44,45]. Previous research has shown that the majority of people are willing to share their personal health data, particularly for research purposes [46].

The proliferation of pervasive computing technologies, such as the “internet of things,” will greatly increase data collection from the personal and private spheres, which may be conducive to social dilemmas such as disempowerment and enclosure. This all the more emphasizes the need to protect the integrity of individuals and their private life, which is key to support the success of such technologies [47]. Legal requirements like the GDPR have strengthened individuals’ rights to the protection of personal data, which, in our opinion, makes patients the only ones who can make sustainable decisions about the sharing of patient-controlled real-world data. Consequently, patients should be in the driver’s seat when these data are utilized to develop, distribute, and evaluate new medical technologies, care processes, and systems. By applying the seven property rights suggested by Ostrom and Hess [25] in this study, rules were specified to protect individuals’ privacy in line with moral and legal requirements. For example, the access, contribution, extraction, removal, and alienation rights to patient-controlled real-world data in the Genia PSS were exclusively specified for the patient and his or her self-determined network. The service providers’ rights were mainly limited to aspects that concern the facility, and its functionalities and use patterns, but no rights to patient-controlled real-world data were specified, which prevents uncontrolled data analytics.

### Applicability to Other Chronic Care Settings

Although the model presented in this study is based on the context of CF care, we believe that it is applicable to other chronic care settings that share similarities with CF care in terms of digital technology use to support coproduction. We selected CF care as the context for this case study because of its strong dependence on coproduction between patients, their families, and health care [20,21]. This type of coproduction is not unique to CF care and we argue that, in light of the demographic shift and technological development that we described in the introduction section of this paper, the transition to co-care in chronic care management [8] is a necessary paradigm shift in the organization and management of health care. Apart from CF care, we have observed other examples of chronic care settings where existing technological, legal, and social structures can be analyzed on the basis of the conceptual model we presented in this study. For example, the round table arena concept has previously been described in the context of rheumatoid arthritis [36]. Similarly, the idea of collaborative chronic care networks stems from research on inflammatory bowel disease [40-42]. Further, the Genia PSS has already spread to other chronic conditions in Sweden (juvenile idiopathic arthritis [28] and acute intermittent porphyria), which allows us to speculate that our model involving three action arenas may be applicable at least in these settings and likely other chronic care settings where co-care is aspired.

Initially, the model can be used to raise questions like “What action arenas can we identify in the chronic care management of a certain patient population/in a certain setting?” “Who are (potential) members of the different arenas?” “Which norms and rules apply in the interactions within and between arenas?” “What real-world data flows exist between arenas?” “What facilities are in use?” and “What property rights have been specified and implemented to protect patients’ privacy and control?” We believe that the discussions triggered by these types of questions may help to uncover some of the social dilemmas that may result from data use and sharing not controlled by the patient, such as disempowerment and enclosure [16]. Such dilemmas may be addressed by formulating and implementing appropriate property rights. In summary, we believe that this model may find application as a tool for the analysis and comparison of chronic care systems in different patient groups and settings, as well as a guide for the development of appropriate technical and legal structures to support coproduction between different arenas.

### Limitations

This study is not without limitations. The model involving three action arenas that was presented resulted from an iterative cycle of development and analysis based on a single case. Because our intention was to present a model that is also applicable in other chronic care settings, we chose a relatively high level of abstraction, compromising the level of detail. We see our model as a general guide rather than a detailed manual on how to manage the sharing of patient-controlled data in chronic care settings. We acknowledge that AH had a central role in both the development and analysis. To maintain neutrality from preconceived ideas or personal interests, the analysis and results have been discussed among all the authors, guided by theory. To further develop the model and test its transferability to other settings, pilot studies have been initiated in the United States (Clinical Trial ID: NCT03910881) and Argentina, and we plan to conduct an international multiple case study.

### Conclusions

To date, little academic research has been devoted to the study of how patient-controlled real-world data can be employed in the introduction and systematic evaluation of novel health technologies. Health care professionals, health care funders, researchers, authorities, and the industry are all dependent on the collection and analysis of real-world data reported by the individual patient whose personal data privacy and control need to be protected. To proactively manage social dilemmas related to shared information and knowledge resources, we suggest the specification of property rights that enable patients to determine how their personal health data are managed and shared in other settings. Our model, developed as a knowledge commons, comprises technical and legal structures that protect the integrity and self-determination of patients in such data sharing to improve individual and population health. Further research is needed to adapt and transfer our proposed knowledge commons model to improve health in other chronic conditions as well.

## Abbreviations

CF: cystic fibrosis
DSS: decision support system
EHR: electronic health record
GDPR: General Data Protection Regulation
PSS: patient support system
